# What proportion of dental care in care homes could be met by direct access to dental therapists or dental hygienists?

**DOI:** 10.1038/sj.bdj.2015.919

**Published:** 2015-12-11

**Authors:** N. P. Monaghan, M. Z. Morgan

**Affiliations:** 1 Dental Public Health, Public Health Wales, Temple of Peace and Health, Cardiff, CF10 3NW; 2 Welsh Oral Health Information Unit, Cardiff University School of Dentistry, Heath Park, Cardiff, CF14 4XY

## Abstract

**Background** Many care home residents require simple dental treatment which is complicated by the need for extra time to deliver dental care. The proportion of their care which could be delivered wholly by hygienists or therapists is unknown.

**Method** 2010 Welsh dental care home survey data on clinical opinion of treatment need and special care skill level required was cross referenced with General Dental Council guidance on direct access.

**Results** Care home residents treatment needs could be wholly addressed by a generalist dental hygienist or therapist for 22% and 27% of cases respectively. With special care experience these figures increase to 43% and 53%.

**Discussion** A large proportion of need in care homes could be wholly provided by hygienists or therapists, especially those with special care experience. The potential efficiency gain of direct access arises from individuals who do not need to see a dentist for any aspects of their care. Direct access to hygienists/therapists for dental care of care home residents should be piloted and evaluated.

**Conclusion** Hygienists and therapists could make a large contribution to addressing dental treatment needs of care home residents and direct access could be an efficient model of care for this setting.

## Background

There is considerable oral disease requiring preventive care, active monitoring and occasional intervention among residents of care homes in Wales^1^,^2^ and in other countries.^3^,^4^,^5^ The UK decennial Adult Dental Health Surveys (ADHSs) commencing in 1968 through to 2009 have reported increasing numbers of older people retaining some natural teeth, even though they are often heavily restored.^6^,^7^,^8^,^9^,^10^ Unfortunately, access to dental care for care home residents is not always straightforward.^11^ Poor access to care means limited opportunity to manage problems by a combination of monitoring, preventive action and intervention. The need for extra time for oral health care among this vulnerable group makes it more expensive than that for the wider population.^12^

Many care home residents require simple dental treatment, complicated by the need for extra time to deliver dental care.^13^ Although there is a large volume of need for improved oral hygiene, scaling of teeth, application of fluoride, and restorations among residents of care homes, relatively little of this need requires a specialist in special care dentistry.^13^ Much of the care only requires a professional with either some special care experience or generalist level experience.^13^ In addition, a considerable proportion of the disease present does not require aggressive interventional treatment.^12^

A Cochrane review of the evidence on the effectiveness, costs and cost-effectiveness of dental auxiliaries in providing care traditionally provided by dentists identified just five studies.^14^ Four of these were over 20 years old, all five were at high risk for bias and no conclusions were drawn from these studies. Recently the General Dental Council (GDC) proposed direct access for patients to members of the dental team without requirement for prior examination, diagnosis and prescription by a dentist.^15^ This is a new development for the UK. Effectively the introduction of direct access changes the position from dentists as gatekeepers of access to dental care to one where a number of members of the dental team are access points.

The proportion of care home residents' care which could be delivered solely by hygienists or therapists is unknown. The items of care which can be undertaken by the various members of the dental team are outlined in the GDC scope of practice guidance.^16^

Data collected in the Welsh dental survey of care home residents included traditional epidemiological measures of disease presence as collected by the ADHS 2009.^10^ This was supplemented by collection of data on the clinical opinion of the examining dentist on:

- likely treatment plan content using a checklist of items of care
- presence or absence of complexity (based on the categories in the BDA special care case mix model^17^
- need for generalist or special care dentistry care.

The treatment plan data were collected before the GDC announced the introduction of direct access to care from dental hygienists and therapists. The scope of practice document identified a list of specified items of care which could and could not be provided by hygienists and therapists. This analysis aims to estimate the proportion of care home residents' dental treatment needs which could be delivered wholly by hygienists or therapists.

## Method

A retrospective analysis of the 2010 survey of Wales care home residents treatment plan related data were cross referenced to the GDC scope of practice document.^16^

Details of the ethical approval, sampling and oral examination are briefly summarised here as they have been previously reported.^12^ The Multi-centre Research Ethics Committee in Wales agreed the study should include residents with and without capacity to consent. Data were collected between October 2010 and June 2011 by 12 examiners (dentists with some experience of special care dentistry) and recorders (dental nurses) working in the Community Dental Services in Wales. Prior to data collection they were trained in the requirements of the Mental Capacity Act 2005, consent, clinical criteria, data entry and adult safeguarding.

In total 228 care homes (residential, nursing, and combined residential and nursing) from 22 local authority areas were randomly selected to participate. Five randomly selected residents from each care home (or all residents where there were five or fewer) were invited to participate. Consent was sought from those residents able to consent. For those without capacity, consent was sought from a person with Lasting Power of Attorney or a Court Appointed Deputy. Participants were free to withdraw from data collection at any point when they felt unwilling or unable to continue.

**Figure 1:**
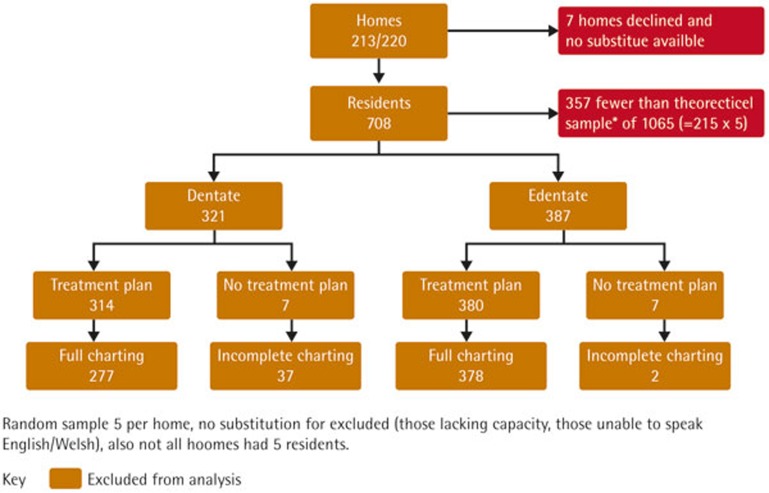
Study participant flow chart

Following each examination and using a checklist of treatments, the dentists were asked to propose a treatment plan to address the pathology identified and commensurate with the difficulty experienced during the examination. They were asked to indicate whether they expected presence or absence of complexity in delivery of that care, including the need for extra time, sedation or general anaesthesia. They were also asked whether that care would need to be provided by a generalist, special-care-experienced- or special-care-specialist dentist. Full dental charting and treatment plan data were collected for 655 residents (Fig. 1).

Details of the examination criteria, treatment plan list, complexity questions and special care experience questions can be found in the survey protocol available from the Welsh Oral Health Information Unit at Cardiff University.^18^

The treatment plan information was collected in sufficient detail and in a format which allowed individual plans to be cross referenced with the GDC scope of practice guidance published in 2013^16^ – see Table 1. This facilitated the identification of treatment plans with content which could potentially be wholly delivered by a hygienist or therapist.

Treatment plans requiring a dentist were filtered out by first identifying those which required a special care specialist or another specialist. Further plans requiring a dentist were filtered out by presence of treatments which could only be provided by a dentist (for example, extraction of permanent teeth). The remaining cases could potentially be cared for by a therapist with special care experience.

Selecting out those cases that a special care therapist could treat but which hygienists cannot (for example, placement of restorations in permanent teeth) left a series of cases who could be cared for by a hygienist with special care experience. Finally for each of these two groups, filtering out cases that required care from someone with special care experience identified those whose care could be wholly provided through direct access by a therapist or by a hygienist without such experience. A similar process was used to identify care home residents whose care could be potentially provided by a clinical dental technician with and without special care experience or extended duties dental nurses.

## Results

Of the 655 care home residents, 22% and up to 27% had treatment needs which could be wholly addressed by a dental hygienist or therapist respectively (Table 2). For hygienists or therapists with special care dental experience the proportions of residents who could have their care needs wholly addressed by a dental hygienist or therapist were 43% and up to 53% (Table 2), respectively. The uncertainty on the upper limit of cases whose care could be provided by a therapist relates to the proportion of restorations that also require endodontic treatment. This is explored further in the discussion section. While dentists with special care experience could provide all aspects of care for 90% of residents, a dentist without such experience could only provide all care for 39% of residents.

The potential role of extended duties dental nurses was so limited in the care home setting that there were no cases where they could wholly provide the treatment plan. The proportions of residents whose treatment needs could be wholly met by a clinical dental technician were 6% for a general technician, and 12% for a technician with special care experience.

With the exception of extended-duty dental nurses, having special care experience typically doubled the percentage of patients whose care needs could be wholly addressed by each dental care professional (Table 2).

## Discussion

There are limitations of this analysis. Dentists were not calibrated in assessing treatment need, so the collective findings are reflective of a range of clinical opinions. The findings are relevant for the UK where the GDC Scope of Practice applies. The data were collected by dentists indicating treatment that would be provided by a dentist with no experience, some experience or specialist ability in special care dentistry. The dentists were not asked to identify which elements of care were appropriate for a hygienist or a therapist. Fortunately, data collection had been in a format which allowed cross referencing of treatment plans with the GDC Scope of Practice.

Data were not specifically collected on endodontic treatment need. Dental therapists can provide restorations in permanent teeth but not endodontic treatment. Dental hygienists cannot provide restorations. The difference in the estimates of work which could be done by therapists but not hygienists relate to restorations (Table 2). In some cases these might have required endodontic treatment. If it is assumed that half of all the individuals requiring restorations also required endodontic treatment (which we consider a high estimate) an estimate of the proportion of 655 cases whose care could be wholly provided by a therapist with or without special care experience would be 48% and 24% respectively. These still constitute a high proportion of care home residents.

Although 90% of care home residents could have their care needs addressed by a dentist with special care experience, and less than 40% by a general dentist, a conclusion that dentists with special care experience are required ignores the potential efficiency gains of direct access. Direct access facilitates alternative models of care where a dentist is not the first point of contact with the dental team. Dentists are an expensive resource and should be deployed on work commensurate with their knowledge and skills and which cannot be delivered by other members of the dental team. The additional potential efficiency gain of direct access arises from individuals who do not need to see a dentist for any aspects of their care.

Given the limited evidence of dental auxiliaries' cost-effectiveness^14^ or of the cost-effectiveness of direct access there is a need for further studies. It is important for patients that there is both good communication and continuity of care if a skill mix team are to be trusted.^19^ Theoretically, direct access is likely to be an efficient model of care where there are large cohorts of individuals with treatment needs within the scope of practice for a hygienist or therapist. Care homes would appear to be an appropriate setting for direct access to therapists or hygienists with special care experience.

Direct access is not currently possible within the terms of the GDS contract without changes in either regulations (England and Wales) or primary legislation (Scotland and Northern Ireland).^15^ It is currently possible to offer direct access from a skill mix team within the community dental services. In areas where there are hygienists and therapists working in care home settings, and therefore having some special care experience, the change to direct access could be considered.

In summary, a significant proportion of care home residents in Wales do not require care from a dentist. A potentially more efficient model would be to have individuals examined first by a hygienist or therapist who is less expensive to employ and is likely to be able to meet all of the care needs for many residents. Direct access to hygienists/therapists for dental care of care home residents should be piloted. Pilots will need to explore both training needs for direct access and training needs in special care dentistry. They will also need to evaluate whether staff are adequately prepared, and the experiences of a range stake-holders of direct access care. Further studies could then explore outcomes of care and of cost-effectiveness.

## Conclusion

Hygienists and therapists could make a large contribution to addressing dental treatment needs of care home residents. Direct access, within a skill mix team, should be piloted to assess the effectiveness and efficiency of such a model of care.

## Commentary

The demography of the UK population is changing in that people are living longer and retaining much, if not all, of their dentition into later life. This paper is timely in that it addresses a number of problems associated with the delivery of care to the residents of care homes, and explores the opportunities that have arisen as a result of direct access to patients without the need for referral by a dentist.

It is recognised that hygienists and therapists are a highly skilled group of professionals, and being granted direct access to patients is clear recognition of their clinical ability and competence. To be in a position to provide care directly to this priority group of elderly and often vulnerable individuals would be a major breakthrough in delivery of their oral care. Hygienists and therapists are able to diagnose and treatment plan within their scope of practice, and have comparable ability with dentists in the recognition of mucosal abnormalities, and appreciate the need to refer to specialists should the need arise.

It has been reported that care home staff are often untrained in oral care with the result that it is sadly neglected, potentially leading to further oral problems and reduced quality of life for their residents. As stated in this paper, much of the routine care required does not need the intervention of specialists or dentists. Oral hygiene, preventive therapies, treatment of periodontal disease and root caries, often exacerbated by xerostomia in the elderly, are all part of the routine care provided by dually qualified hygienist-therapists, as most graduates are today. There is also the ticking time bomb of those with complex restorative work, such as implants, who require high-quality maintenance and disease control.

Hygienists and therapists working in the community or public dental service are often heavily involved in special care dentistry, and are, therefore, experienced in care of the elderly, among other priority groups. In addition to clinical expertise, it is often patience, care and compassion which is required to treat elderly patients appropriately. If NHS list or provider numbers were allocated to hygienists and therapists, it would allow greater access for patients. In addition to this requirement, the removal of the restriction on the application of fluoride and the administration of local analgesia would enable these clinicians to exercise their full scope of practice in a care home setting. Surely this is an obvious course of action for hygienists and therapists to be used to their full potential, placing them in a position to address the unmet oral needs of this increasingly significant patient group.

**Margaret K. Ross Senior Lecturer for Dental Care Professionals Edinburgh Dental Institute, University of Edinburgh**

## Author questions and answers

**1. Why did you undertake this research?**

We knew from historical studies that care home residents had poorer oral health than peers in the community and had difficulty accessing dental care. We also knew that care home residents are increasingly dentate. We collected the data to compare the current oral health status of care home residents with peers examined in the Adult Dental Health Survey. Our previous analyses of this data showed that simple treatment need was complicated by requirement for special care experience in many cases. After we collected this data direct access was introduced. We recognised that the care needs might be appropriate for hygienists and therapists and wished to quantify the proportion of work which they might undertake without the need to see a dentist.

**2. What would you like to do next in this area to follow on from this work?**

The dental care needs of a more dentate care home population are increasingly complex – partly due to the presence of heavily restored teeth and partly due to co-morbidity which complicates delivery of care. We would encourage others to develop dental epidemiology for our increasingly dentate older population. Summaries of the findings will inform both policy at Welsh Government level (and possibly beyond Wales) and the commissioning of preventive interventions and NHS dental services for care home residents.

We intend to, and hope that others across the UK will also, secure the necessary resources to pilot and evaluate the introduction of direct access therapist and hygienist services in care homes. These should be delivered as part of a wider team including specialists in special care dentistry and special care experienced dentists.

## Figures and Tables

**Table 1:** Scope of practice

Element of care	Hygienist	Therapist	Dentist	Clinical dental technician
Examination	Y	Y	Y	N
Radiograph/s	Y	Y	Y	N
OHI (to the resident or carer)	Y	Y	Y	N
Sub &/or supra-gingival debridement	N	Y	Y	N
Filling/s	N	Y	Y	N
Simple extraction/s	N	N for permanent	Y	N
Copy dentures	N	N	Y	For complete dentures only
New denture/s, not copy F	N	N	Y	For complete dentures only
Denture adjustment/repair	N	N	Y	For complete dentures only
Soft tissue minor oral surgery	N	N	Y	N
Hard tissue minor oral surgery	N	N	Y	N
Sealing of root/s	Y	Y	Y	N
Supplemental fluoride	Y	Y	Y	N
Sedation*	N	N	Y	N
General anaesthesia	N	N	Y	N
Other treatment	N	N	Y	N

^*^With additional training hygienists and therapists can treat patients using inhalational sedation

**Table 2:** Skill mix requirements of care home residents examined in Welsh dental care home survey 2010–11

% of 655 residents who potentially could be managed by:		
Dental team member	Generalist	Special care experienced
Dentist	38.7	89.6
Clinical dental technician	5.7	11.5
Therapist	26.8	52.5
Hygienist	21.9	43.2

^*^No residents could have all their care provided by an extended duties dental nurse

## Notes

**Refereed Paper**
